# StUdy of Gestational diabetes And Risk using Electronic Data (SUGARED): a population-based cohort study—study protocol

**DOI:** 10.1136/bmjopen-2024-087248

**Published:** 2024-12-26

**Authors:** Deborah Randall, Ibinabo Ibiebele, Tanya Nippita, Siranda Torvaldsen, Jonathan M Morris, Felicity Gallimore, Tessa L Weir, Sarah Glastras

**Affiliations:** 1 Reproduction and Perinatal Centre — Northern Precinct, Faculty of Medicine and Health, The University of Sydney, Sydney, New South Wales, Australia; 2 Clinical Excellence Commission, NSW Health, St Leonards, New South Wales, Australia; 3 Kolling Institute, Northern Sydney Local Health District, St Leonards, New South Wales, Australia; 4 Department of Obstetrics and Gynaecology, Royal North Shore Hospital, St Leonards, New South Wales, Australia; 5 Department of Diabetes and Endocrinology, Nepean Blue Mountains Hospital, Kingswood, New South Wales, Australia; 6 Department of Diabetes, Endocrinology & Metabolism, Royal North Shore Hospital, St Leonards, New South Wales, Australia

**Keywords:** Diabetes in pregnancy, EPIDEMIOLOGY, OBSTETRICS, Pregnant Women, STATISTICS & RESEARCH METHODS

## Abstract

**Abstract:**

**Introduction:**

The incidence of gestational diabetes mellitus (GDM) in Australia has tripled in the last 20 years. Consequently, over 40 000 pregnancies are now diagnosed as ‘higher risk’ each year. This has increased antenatal surveillance and obstetric intervention, often in the form of delivery earlier than 39 weeks gestation. The StUdy of Gestational diabetes And Risk using Electronic Data (SUGARED) project aims to use large population-based and routinely collected linked health data to (1) personalise risk prediction of adverse pregnancy outcomes for women undergoing glucose tolerance testing, (2) guide optimal birth timing for women with diet-controlled GDM and (3) examine variation in GDM management and pregnancy outcomes in New South Wales (NSW), Australia.

**Methods and analysis:**

This retrospective cohort study using linked, routinely collected health data includes all women who gave birth from January 2016 to December 2020 in NSW. The cohort will include approximately 475 000 pregnancies, with >70 000 diagnosed with GDM. The study will link birth data to hospital data and birth/death registry data. In addition, clinical pathology results and detailed clinical information from a subset of public hospital pregnancies in 13 of 15 area health services will be linked. To address the three main aims, we will use statistical methods including logistic regression and K-fold cross-validation for risk prediction, a propensity-score matching ‘target trial’ method to examine birth timing, and multilevel modelling to examine hospital variation.

**Ethics and dissemination:**

Ethics approval for the study has been granted by the NSW Population and Health Services Research Ethics Committee. We will communicate evidence generated from SUGARED to the local health districts and their clinicians, as well as potentially optimising dissemination using existing digital infrastructure.

**STRENGTHS AND LIMITATIONS OF THIS STUDY**

- The StUdy of Gestational diabetes And Risk using Electronic Data project will use a large population-based cohort of pregnant women with and without gestational diabetes mellitus (GDM), providing a large sample size to investigate evidence gaps in the diagnosis, treatment and management of GDM in pregnancy.
- In novel data linkage, detailed clinical and pathology fields will be linked with the routinely collected perinatal and hospital data so that analyses can include such measures as glucose tolerance tests and whether the GDM is treated with diet or medication.
- A K-fold approach will be used to derive and validate risk prediction models, identifying subgroups of women with GDM who are at higher or lower risk of adverse outcomes.
- A ‘target trial emulation’ approach to determine the optimal timing of birth for women with GDM will be used, comparing pregnancies with planned delivery versus expectant management.
- The main limitation of this study is the observational nature of the data, meaning that the pregnancies and their outcomes are observed under normal treatment, and therefore, the ability to determine causal associations will be difficult. However, there is variation in diagnosis, treatment and management of GDM pregnancies across the New South Wales treatment centres, and this variation can be used to assess differences in the natural ‘treatment’ and ‘comparison’ groups.

## Introduction

 Gestational diabetes mellitus (GDM) is defined as glucose intolerance first diagnosed in pregnancy. Women with GDM are at increased risk of short-term pregnancy complications, obstetric interventions^1^ and longer-term development of type 2 diabetes and cardiovascular disease.^2^ Children of mothers with GDM face increased risk of preterm birth, higher birth weights, admission to neonatal intensive care, hypoglycaemia, respiratory distress^1^ and adverse health outcomes as adults.^2^ Australian women with GDM are often moved into a ‘higher risk’ care model, with more appointments, monitoring and testing than other pregnant women. They are recommended to adhere to lifestyle advice, undergo frequent blood glucose monitoring and, in some cases, require medication and undergo obstetric intervention such as labour induction.^3^

GDM incidence in Australia has tripled from 5.2% in 2000*–*2001 to 16.1% in 2017*–*2018,^4^ partly due to changes in maternal age, obesity and ethnicity but mostly due to altered diagnostic criteria identifying a greater proportion of women with GDM.^5^,^7^ Over 40 000 pregnancies are now classified annually as ‘higher risk’ in Australia, increasing antenatal surveillance and obstetric intervention, often in the form of delivery earlier than 39 weeks gestation. Though some women with severe or poorly controlled GDM have higher perinatal risks,^8^ many women require minor lifestyle changes, if any, and have very good perinatal outcomes. We posit that a universal set of GDM diagnostic criteria and management plan unnecessarily raises the obstetric intervention rate and cost burden on the healthcare system.^9^

The previous Australian diagnostic criteria were based on predicting type 2 diabetes risk after pregnancy.^10^ It was a two-step process, in which all pregnant women undertook a non-fasted glucose challenge test, with only those reaching a blood glucose threshold completing a subsequent 75 g oral glucose tolerance test (OGTT). The 2008 Hyperglycaemia and Adverse Pregnancy Outcomes study found evidence of a continuous relationship between maternal glucose levels and adverse outcomes in women with blood glucose levels below those diagnostic of GDM at the time.^5^ Those results led to new diagnostic criteria, endorsed by the International Association of Diabetes and Pregnancy Study Groups (IADPSG) in 2010, the WHO and Australasian Diabetes in Pregnancy Society in 2013, and recommended for adoption by the Royal Australia and New Zealand College of Obstetricians and Gynaecologists from January 2015.^11^,^13^ The new criteria used new glucose diagnostic cut-offs (fasting plasma glucose≥5.1 mmol/L, 1-hour plasma glucose≥10 mmol/L and 2-hour plasma glucose≥8.5 mmol/L^11^) and changed the diagnosis of GDM to a one-step universal fasted 75 g OGTT.

Single-centre observational studies in Australia have found no evidence of improved maternal or neonatal outcomes after the diagnostic change but noted increased GDM incidence^14^ and hospital costs.^15^ Even more compelling, a clinical trial comparing two-step Carpenter-Coustan screening (recommended by the American College of Obstetricians and Gynecologists (ACOG) and similar to the previous Australian two-step approach) with the revised IADPSG one-step approach found a difference in the proportion of women diagnosed (two-step: 8.5%, one-step: 16.5%) but unchanged perinatal and maternal complication risks. Similarly, the Gestational Diabetes Mellitus Trial of Diagnostic Detection Thresholds (GEMS) trial found a large difference in the proportion of women identified as having GDM (6.1% vs 15.3%) but a negligible difference in the proportion in each group with large-for-gestational age babies in women diagnosed and treated by either stringent or more relaxed GDM diagnostic criteria.^16^

Due to scarce and somewhat conflicting data, there are varying Australian and international recommendations for birth timing in women with GDM. The UK National Institute for Health and Care Excellence (NICE) guidelines recommend women with GDM and no complications deliver by 40^+6^, but that earlier birth be considered for women with GDM and other complications.^17^ The ACOG recommends that delivery in women with diet-controlled GDM should not be <39 weeks, unless otherwise indicated, and there should be expectant management up to 40^+6^ weeks.^18^ Within Australia, Queensland and South Australian guidelines recommend that women with well-managed, diet-controlled GDM await spontaneous labour.^19 20^ While many guidelines recommend expectant management, unless there are relevant clinical factors favouring early delivery, there is little consensus regarding the relevant clinical factors that should favour delivery before 39 weeks between states and centres and include a combination of macrosomia, foetal growth patterns, polyhydramnios, oligohydramnios, glycaemic control, maternal obstetric history, high body mass index (BMI), parity, cervical status and/or the woman’s preference. With the increase in number of women diagnosed with GDM, there is concern over a subsequent increase in early planned births and obstetric intervention.

A wealth of prospectively recorded data, generated through routine hospital care, can be linked at a population level and can deliver insights and guide clinical decision-making about antenatal management and timing of birth. Compared with a randomised controlled trial, using routinely collected clinical data has the advantage of examining patients in routine clinical practice, and significant variation in clinical practice across clinicians and hospitals can be used to assess outcomes. Through innovative use of routinely collected data and leading statistical methods, the StUdy of Gestational diabetes And Risk using Electronic Data (SUGARED) project objectives are to devise a more personalised risk prediction for the thousands of women with GDM, address the gap in evidence around birth timing for GDM pregnancies and examine variation in the management of and outcomes for GDM pregnancies. SUGARED will be conducted in New South Wales (NSW), Australia, using existing health data linkage infrastructure to create a population-level cohort of pregnancies to which we will link hospital, emergency, clinical maternity, pathology and vital status data.

To address the existing evidence gaps in the diagnosis and management of GDM, the SUGARED project aims to:

1. Identify subgroups of women with GDM with higher and lower risk of adverse outcomes.
2. Among women with diet-controlled GDM, provide a gestational age-specific risk of neonatal morbidity and perinatal mortality associated with planned birth (induction of labour or no-labour caesarean section) compared with expectant management at term (≥37 weeks).
3. Identify state-wide hospital variation in GDM-related risk, treatment and outcomes, including contributing individual and hospital-level factors, and the impact of the treatment variation on outcomes for women with GDM.

The results of aim 1 will enable obstetricians to better target obstetric surveillance and intervention(s). Aim 2 findings will provide guidance on the optimal timing of birth for women with GDM. The results from aim 3 will provide an understanding of the hospital variation in obstetric management for women with GDM and how this variation influences maternal and neonatal outcomes. This can then be used to facilitate a reduction in variation regardless of area of residence, leading to optimal outcomes for women with GDM.

## Methods and analysis

### Study design and population

This is a retrospective cohort study using linked routinely collected health data. All women who gave birth in NSW from January 2016 to December 2020 are included. The cohort will comprise approximately 475 000 pregnancies, with >70 000 diagnosed with GDM.

### Data sources

**Figure 1:**
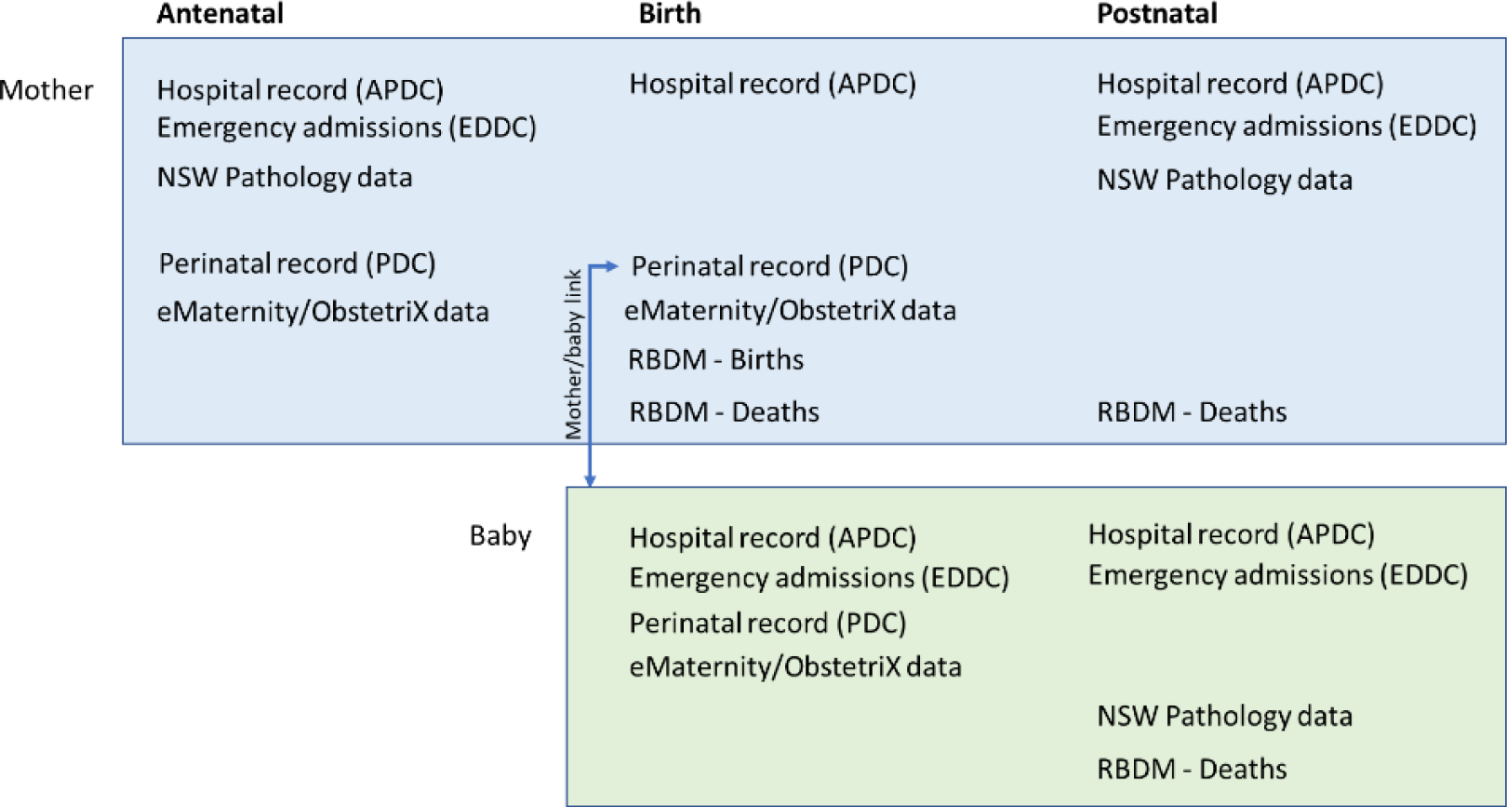
Population-based linkage of maternal antenatal, birth and postnatal data, including hospital, perinatal, pathology and deaths, and linked infant perinatal, hospital, pathology and death data. APDC, Admitted Patient Data Collection; EDDC, Emergency Department Data Collection; NSW, New South Wales; PDC, Perinatal Data Collection; RBDM, Register of Births, Deaths and Marriages.

The NSW Perinatal Data Collection (PDC) will form the base of our cohort and be linked with other datasets, as shown in figure 1.

The PDC is a statutory collection and primary source of information about pregnancy and outcomes for private, public and home births in NSW (≥20 weeks gestation or ≥400 g birth weight). It includes data on maternal demographics, pregnancy, delivery (mode and indication) and outcomes, such as gestational age and birth weight. Births from January 2016 to December 2020 will form the study population, and linked births from January 2001 to December 2015 will contribute previous birth information for cohort mothers. The NSW Register of Births, Deaths and Marriages (RBDM) is another statutory data collection that enumerates births. The RBDM birth data (July 2001 to December 2021) will provide additional demographic information for both parents and linked births, and the RBDM death data (January 2016 to December 2021) will provide linked death information for cohort mothers and babies.

The NSW Admitted Patient Data Collection (APDC) is a census of all inpatient hospital discharges. It will provide linked records for mothers and infants. It has demographic, clinical and health service information for each hospital separation. Up to 55 diagnoses and procedures are recorded for each hospital admission and coded according to the International Statistical Classification of Diseases (ICD) and Related Health Problems, Tenth Revision, Australian Modification and the Australian Classification of Health Interventions. Cohort mothers will have linked inpatient hospital episodes from July 2001 to December 2021 to provide lookback information about chronic conditions, previous procedures and health service usage, as well as antenatal and postnatal conditions and outcomes. Cohort babies will have linked inpatient episodes from January 2016 to December 2021 to provide information on neonatal morbidity and procedures, as well as any readmissions in the first year of life.

The NSW Emergency Department Data Collection (EDDC) collates patient presentations to the emergency departments (EDs) of public hospitals and in-scope contracted private hospitals in NSW, including presentations to 184 hospitals in NSW from 2016 to 2017. It includes data on patient demographics, triage category and diagnosis coded to ICD or Systemized Nomenclature of Medicine, Clinical Terms. All ED presentations from January 2005 to December 2021 will be linked with the cohort mothers, and ED presentations from January 2016 to December 2021 will be linked with the cohort babies to provide information on presentations, conditions and health service usage.

The NSW Pathology Data contains pathology test results from NSW public pathology facilities. Relevant pathology test results for the cohort mothers and babies from January 2016 to December 2020 will be linked to the cohort to get more information detail on OGTT results for the mother and blood glucose results for the mother and baby after the birth.

**Figure 2:**
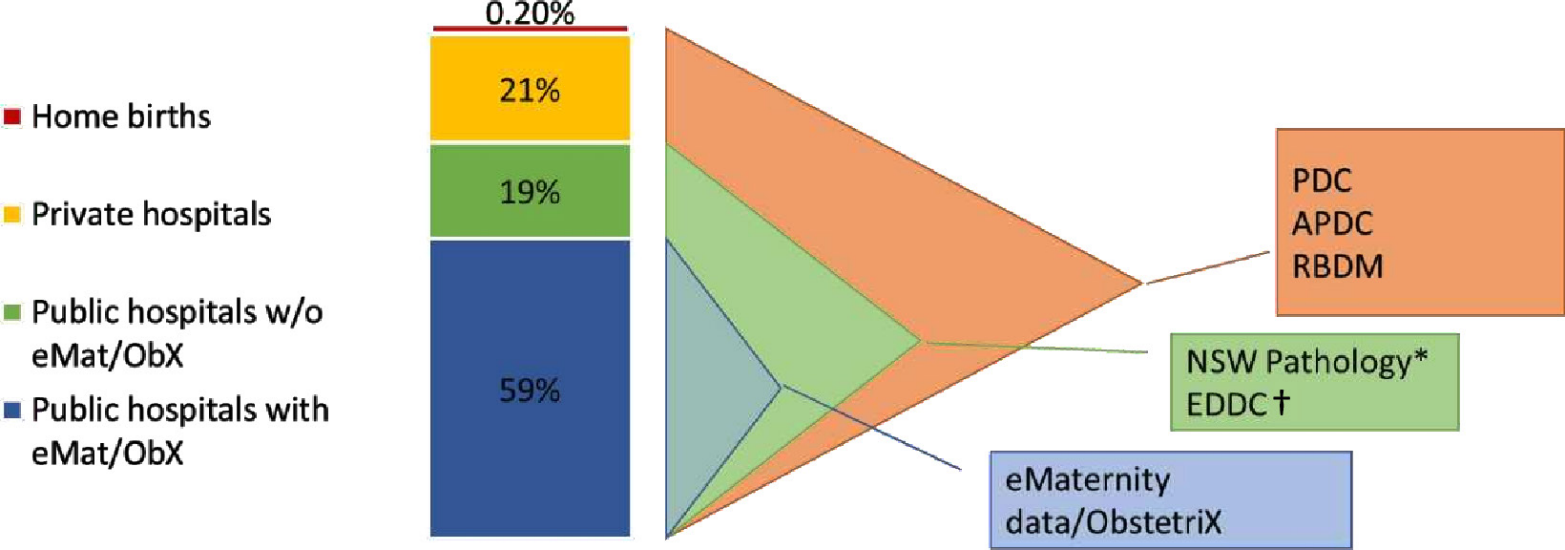
Estimated NSW birth population coverage with the different linked datasets. *NSW Pathology contains only pathology tests done in public hospital pathology laboratories. †Emergency admission (EDDC) covers most public ED patients, but not the entire NSW population. Source: NSW Mothers and Babies Report, 2018. APDC, Admitted Patient Data Collection; EDDC, Emergency Department Data Collection; NSW, New South Wales; PDC, Perinatal Data Collection; RBDM, Register of Births, Deaths and Marriages.

The eMaternity/ObstetriX data system (clinical maternity data) holds information about pregnancy and births in public hospitals in 13 out of 15 NSW local health districts (LHDs). During the study period, these LHDs transitioned from ObstetriX to eMaternity. There was a 6-month system overlap in each LHD, but all were using eMaternity alone by the end of 2017. A minimum dataset from eMaternity/ObstetriX is sent to NSW Health, collated into the PDC, along with the same information from private hospitals, the remaining public hospitals and some independent midwives. The clinical maternity data will be linked with the cohort for a small number of additional variables not in the PDC, but important for the understanding of variation in risk, diagnosis, severity and management of GDM, such as family history of diabetes and type of diabetes therapy during pregnancy. This more detailed maternity data will be linked with the population-level data from January 2016 to December 2020. The estimated NSW birth population coverage linked with each of the additional datasets is shown in figure 2.

### Data linkage

All record linkage will be conducted by the NSW Centre for Health Record Linkage (CHeReL)^21^ in accordance with NSW privacy guidelines. Probabilistic matching methods will be used to link individuals, incorporating name, date of birth, sex and address information, via ChoiceMaker software. The CHeReL uses the best-practice separation principle, wherein identifying information from each dataset is separated from clinical content data before linkage.^22^ Once linked, each record is assigned a unique person-number, with linkage keys identifying mother and baby pairs. Unique person-numbers are then attached to clinical data by data custodians, and deidentified health data are provided to researchers who merge datasets using the person-number. CHeReL record linkage validity is extremely high, with <1% of records having incorrect matches.^23^

### Defining gestational and pre-existing diabetes

The population with and without diabetes will be defined from the PDC and APDC. From 2016, the PDC revised diabetes capture, distinguishing pre-existing type 1 and type 2 and GDM. The APDC captures diabetes type in the antenatal period (if admitted) or in the birth record. Lookback information for the PDC and APDC will be used to identify women recorded as having GDM, but with pre-existing diabetes recorded in hospital or previous birth records. A previous validation study examining the reporting of diabetes in pregnancy in the PDC and APDC found better recording in hospital data, and as such, both linked datasets will be used to define the diabetes and diabetes type.^24^ Degree of glucose tolerance will be defined using OGTT results from Pathology and clinical maternity data. Whether the diabetes was diet or medication-controlled is recorded in the clinical maternity data.

### Outcome variables

Outcome measures will include as follows:

1. Obstetric interventions.
  - Caesarean section.
  - Induction.
  - Caesarean or induction<39 weeks gestation.
  - Instrumental birth.
  - Episiotomy.
2. Maternal outcomes.
  - Maternal morbidity (validated composite indicator).
  - Pregnancy hypertension.
  - Pre-eclampsia.
  - Postpartum haemorrhage.
  - Perineal tears.
  - Blood transfusion and number of blood packs used.
  - Blood glucose at 6 months after birth.
  - Length of hospital stay.
3. Infant outcomes.
  - Preterm birth (spontaneous).
  - Stillbirth.
  - Neonatal death.
  - Perinatal mortality.
  - Neonatal morbidity (validated composite indicator).
  - Apgar at 5 min.
  - Birth weight.
  - Large for gestational age.
  - Small for gestational age.
  - Baby’s length.
  - Shoulder dystocia.
  - Bone fracture.
  - Arm/hand palsy related to birth injury.
  - Neonatal cord-blood pH.
  - Transfer to neonatal intensive care or special care nursery.
  - Hypoglycaemia.
  - Neonatal jaundice.
  - Blood glucose in first 6 weeks postpartum.
  - Length of hospital stay.
  - Hospital admissions in the first year of life.
  - Mortality within the first year.

### Explanatory variables

Individual explanatory variables will include as follows:

1. Demographic and socioeconomic factors:
  - Maternal age.
  - Country of birth.
  - Aboriginality.
  - Residence remoteness, calculated using Statistical Area 2 of residence (SA2), the Australian Bureau of Statistics (ABS) remoteness classification for Statistical Area 1 and the population-weighted correspondence to SA2.
  - Area-level socio-economic status (using the ABS Socio-Economic Indices for Areas classification for SA2).
2. Maternal risk factors:
  - Maternal height, weight and BMI.
  - Previous birth outcomes.
  - Parity.
  - Smoking in pregnancy.
  - History of endocrine, metabolic and gynaecological conditions.
  - Chronic conditions and previous surgery.
3. Pregnancy risk factors:
  - Plurality.
  - Gestational hypertension and pre-eclampsia.
  - Assisted reproductive technology.
  - Antenatally identified foetal growth restriction and macrosomia.
  - Antenatal electrolytes and results of liver function tests, full blood count coagulation studies and urine tests.
  - Antenatal medical treatment of GDM (insulin, oral hypoglycaemic medication).
4. Health service use:
  - Birth hospital.
  - Antenatal care information.
  - Model of care.
  - Antenatal ED and hospital admissions.
5. Labour and birth characteristics:
  - Labour onset, induction and augmentation of labour.
  - Labour interventions.
  - Mode of birth.
  - Pain relief.
  - Gestational age.

### Analysis plan: risk prediction models (aim 1)

**Figure 3:**
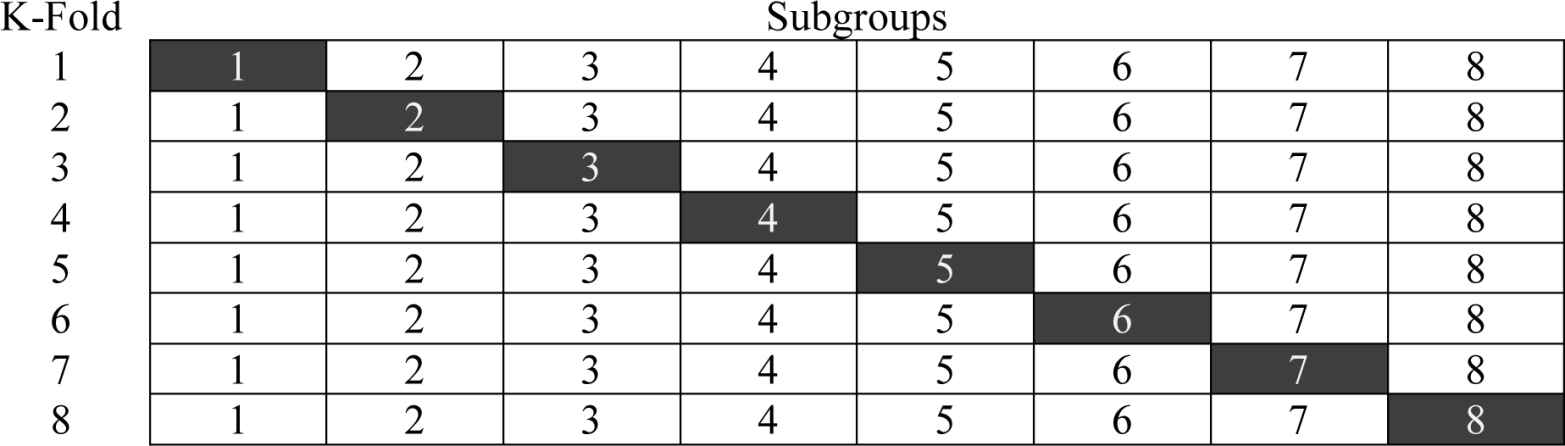
Derivation and validation cohorts for each of the eight folds.

A K-fold approach will be used to derive and validate risk prediction models identifying groups of women with GDM who are at higher or lower risk of adverse outcomes. The study population, comprising all women in the cohort who have been identified as having GDM, will be randomly divided into roughly equal subgroups balancing prediction error and computational time. In each fold, one subgroup will be a validation cohort, and the other subgroups will be combined as the derivation cohort. Figure 3 provides an illustration of the k-fold approach using an 8-fold as an exemplar of the methodology, where the whole population gets randomly assigned to eight roughly equal groups. Once assigned, individuals remain in their allocated group. A ‘fold’ refers to a round of analysis where the remaining groups are combined to generate the risk prediction model while the validation cohort is then used to validate it. In an eightfold, these analyses are run eight times, with each group being used as the validation cohort once and as part of the derivation cohort seven times.

Using the derivation cohort, univariate repeated measures logistic regression modelling will assess the relationship between the primary composite outcome (any of the following: large for gestational age, stillbirth, neonatal death, shoulder dystocia, bone fracture, arm/hand palsy related to birth injury, gestational hypertension or pre-eclampsia) and explanatory variables. Repeated measures logistic regression will take into account the non-independence of two or more pregnancies to the same woman during the study period. Two-way interactions (eg, BMI and hyperglycaemia) will be examined, and the collective and individual effect on the multivariate model assessed. Multivariable repeated measures logistic regression will estimate adjusted ORs and their 95% CIs.

Using the validation cohort, agreement between the predicted and observed probabilities of the primary composite outcome will be assessed by Hosmer-Lemeshow goodness of fit testing.^25^ Ability of the prediction model to differentiate high-risk and low-risk women will be assessed using area under the receiver operating characteristics curve.^26^ The sensitivity, specificity, negative and positive predictive values will be calculated for each validation cohort.

### Analysis plan: gestational age-specific risk associated with planned birth (aim 2)

**Figure 4:**
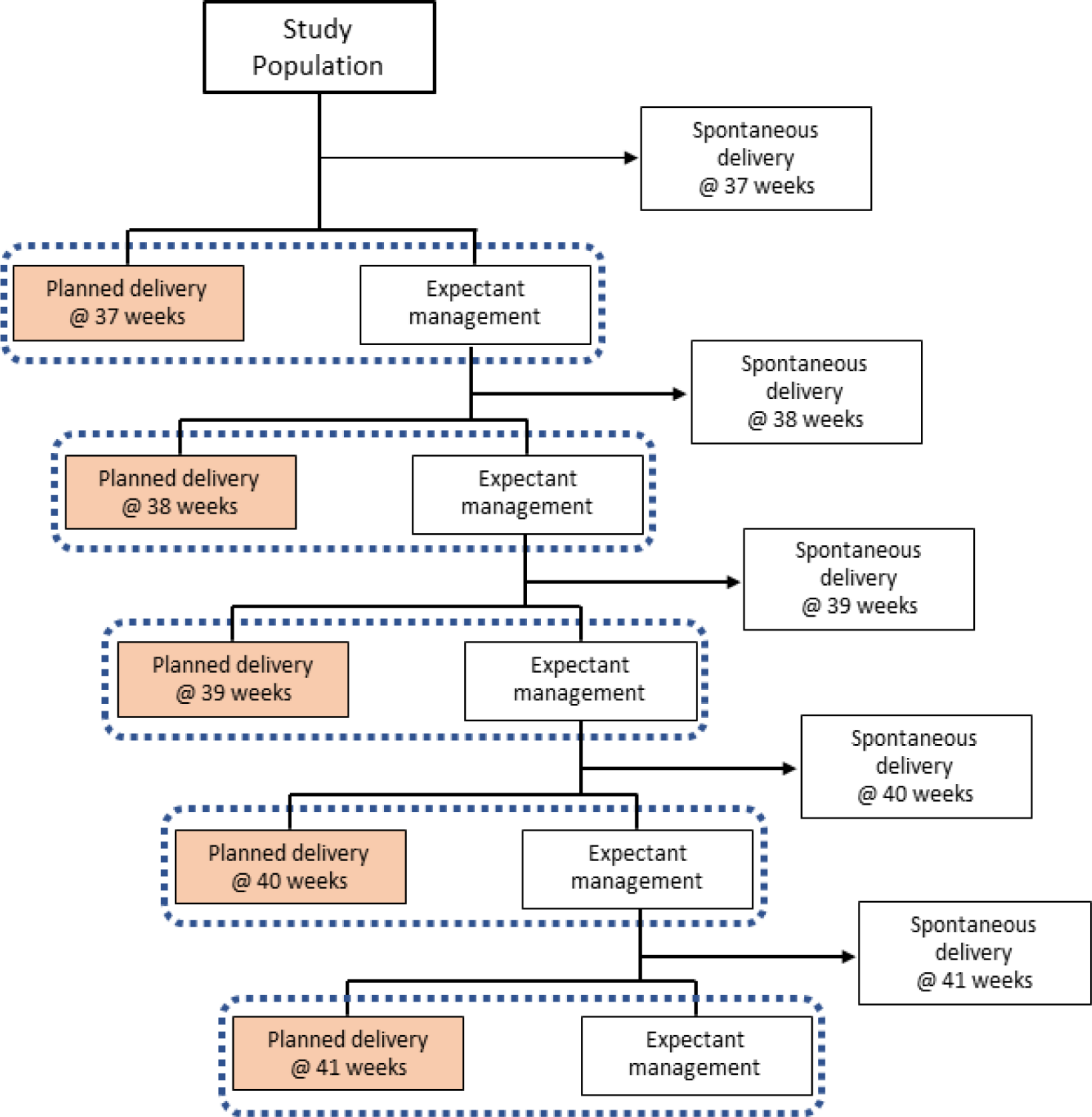
Study population flowchart. Dashed boxes indicate where propensity score matching will be used to select controls from the expectant management group.

In order to provide guidance to clinicians on the optimal timing of birth for women with GDM, we will take a ‘target trial emulation’ approach.^27^ The gestational age-specific risk of neonatal morbidity and perinatal mortality will be assessed for planned birth at 37*–*41 weeks’ gestation inclusive. At each gestational week of interest, pregnancies with planned delivery will be compared with pregnancies with expectant management using multivariable logistic regression. Inverse probability weighting will be used to create exchangeability between the delivered and expectantly managed groups (figure 4). The study population for this research question will be restricted to women with diet-controlled GDM with a singleton pregnancy and cephalic presentation. Women with pre-existing comorbidity will also be excluded.

### Analysis plan: hospital variation (aim 3)

The hospital variation analyses will examine outcomes for mothers within hospitals. Multilevel modelling takes the hierarchical structure of patients within hospitals into account, quantifying the degree that outcome variation is due to patient characteristics or hospitals.

To make meaningful comparisons between hospitals, an adjustment for different levels of risk in pregnant women in each hospital will be made, using information from the routine datasets (eg, maternal and pregnancy characteristics). Then, unexplained hospital variation in obstetric management and outcomes of pregnancies with GDM will be identified and quantified with the variance partitioning coefficient and, for logistic regression models, the median OR.^28^ The multilevel analyses with single outcomes will be extended to multivariate multilevel models to test the association between obstetric management practices, such as early planned births, and health outcomes.^29^ To understand causal effects (eg, the causal impact of induction of labour on risk of caesarean section among women with GDM), directed acyclic graphs will be used, thereby avoiding over-adjustment and collider bias.^30^ These analyses will also be undertaken within a ‘target trial’ framework, where the exchangeability of treatment groups is emulated through adjustment, eligibility criteria match the hypothetical trial and eligibility criteria are met at or before time zero.^31^

### Missing data

Missingness extent and mechanisms will be determined in all instances, and imputation used to reduce bias and maximise data efficiency.^32 33^ Multiple imputation models will consider clustered data structure.^34^ Where multiple imputation is impractical or there is residual bias, quantitative bias analysis will be used to understand the implications of missing data.^33^

### Data quality

Although use of population-linked data is a strength of this study, there are limitations with the use of surveillance and administrative health data. Australian and international studies have reported variable accuracy for diagnoses, procedures, pre-existing conditions and outcomes from routinely collected perinatal and hospital data.^35^ Reassuringly, specific validation studies, including our own, for the PDC (birth data) and APDC (hospital data) show excellent levels of agreement with the medical record, low rates of missing data and high accuracy for reporting of diagnoses and procedures during labour.^36^,^39^ Our group has also validated the recording accuracy of pre-existing diabetes and GDM in the PDC and APDC against medical records and found that recording of these conditions in the APDC data was more accurate than in the PDC data.^24^ Our study will use these findings to inform ascertainment of GDM in pregnancy. We will also use data linkage across datasets to determine the reporting consistency of major conditions and demographic factors across routinely collected data and publish these findings to assist others using the same datasets.

### Patient and public involvement

This study was informed by feedback from women with GDM who indicated feelings of anxiety, lack of control and confusion after their diagnosis. We intend to convene a consumer reference group prior to developing data tools and educational materials to ensure that they are informed by women’s experiences and views. The group will receive regular project communications in order to maintain their engagement in the study, with input sought at the start of the project and at regular time points throughout, including focus group feedback on the education resources.

### Ethics and dissemination

The study has been approved by the NSW Population and Health Services Research Ethics Committee (project identifier 2020/ETH02737).

We intend to disseminate evidence generated from the SUGARED project through data tools and educational materials. Data tools may include dynamic model-based risk prediction tools, available to clinicians during their consultations with pregnant women with GDM, as an additional resource to add context to the current diagnostic criteria and to improve shared decision-making. Shared decision-making is recognised as good clinical practice and an ethical obligation, aimed at improving health outcomes and reducing health costs.^40^ In Australia, shared decision-making has been embraced to reduce unwarranted healthcare variation and drive appropriate care. Evidence shows improved patient satisfaction with care, and that better-informed patients make more conservative, less costly treatment choices due to having a more realistic appreciation of the likely benefits and risks.^41^ Our research group has previously produced educational materials for birth timing decisions (www.everyweekcounts.com.au). We plan to produce similar materials for SUGARED findings. The findings will be translated into easily understood infographics, giving pregnant women agency over pregnancy decisions, and providing a reliable information resource that clinicians can use to assist in the consultation process.

Our findings on hospital variation in GDM-related risk, treatment and outcomes will be communicated to the LHDs and their clinicians. One option for information dissemination could be the use of existing digital infrastructure such as the Quality Improvement Data System (QIDS), QIDS MatIQ^42^ developed by our partner organisation, the NSW Clinical Excellence Commission.

## Conclusion

Strong evidence and guidance for clinicians managing pregnancies with GDM is lacking. The current diagnostic criteria identify more than 15% of pregnancies in Australia with GDM, yet there is no clear evidence that this increased identification of GDM improves outcomes. The SUGARED project will fill the evidence gaps by using routinely collected and linked health data in novel and principled ways, led by our multidisciplinary team including obstetricians, endocrinologists, epidemiologists, biostatisticians, health informaticians and data scientists, with clinical, domain and linked data expertise. The study will leverage the leading population-wide linked data resource in NSW, Australia, and for the first time, link additional clinical fields to better match the data available to clinicians when they are making management and birth timing decisions for women with GDM.
