# Supplementary material for: StUdy of Gestational diabetes And Risk using Electronic Data (SUGARED): a population-based cohort study—study protocol
Source: BMJ Open. 2024 Dec 26;14(12):e087248. doi: 10.1136/bmjopen-2024-087248 (PMC11683978; doi:10.1136/bmjopen-2024-087248)
Supplement: Supplementary file 1 [file bmjopen-2024-087248.reviewer_comments.pdf]

PEER REVIEW HISTORY

BMJ Open publishes all reviews undertaken for accepted manuscripts. Reviewers are asked to complete a checklist review form and are provided with free text boxes to elaborate on their assessment. These free text comments are reproduced below.

ARTICLE DETAILS

Title (Provisional)

Study of Gestational Diabetes and Risk using Electronic Data (SUGARED), a population-based cohort study – Study Protocol

Authors

Randall, Deborah; Ibiebele, Ibinabo; Nippita, Tanya; Torvaldsen, Siranda; Morris, Jonathan M; Gallimore, Felicity; Weir, Tessa L; Glastras, Sarah

---

VERSION 1 - REVIEW

**Reviewer**	**1**
**Name**	**Christensen, Maria Hornstrup**
**Affiliation**	**Odense University Hospital**
**Date**	**23-Apr-2024**
**COI**	**I have no competing interests**

General comment

The study protocol represents a thorough and ambitious piece of work. The study will contribute with novel, highly relevant and valuable insights for clinical practice that will further qualify and optimise the shared decision-making and care for women with GDM and their offspring. As such, I anticipate that the results from the study will be eagerly awaited by all involved in GDM clinical management as well as in GDM research.

The interdisciplinary author group constitutes an important strength for the designing and the conduct of the study and must be praised.

I have the following specific comments and questions to the study protocol:

• For clarification, I suggest that the abstract states that Aim 2 addresses women with diet-controlled GDM (as noted on page 10). In addition, I recommend that it is mentioned in the overview of the aims on page 5.

• How come the study period is defined as 2016–2020? What is the background for not extending the study period to e.g. 2022/2023?

• The concept regarding the study population for Aim 1 is not completely clear to me. Does the study population comprise all women both with and without GDM; women undergoing glucose tolerance testing (page 2, l. 23); or women with GDM (page 5, l. 46; page 9, l. 43)? If non-GDM women are included in the study population, how will it be dealt with that 'control women' may have pregestational diabetes (type 1, type 2, other types) and hence contribute with data to the non-GDM group?

• How will the possibility of additional pregnancies by each individual woman during the study period be handled? And if the study unit is pregnancy (rather than the individual woman), how will this be accounted for with regard to interdependent data?

• The primary outcome contains adverse outcomes representing very different levels of seriousness (any of: LGA, stillbirth, neonatal death, shoulder dystocia, bone fracture, arm/hand palsy related to birth injury, gestational hypertension or pre-eclampsia). Additionally, 6 of them are primarily related to the offspring and 1 primarily related to the mother. I wonder what the rationale for choosing the specific outcomes included in the primary outcome was and I have some concerns that the interpretation of this outcome will be diffuse and difficult and offer knowledge with somewhat questionable clinical applicability? I would like to hear the authors' thoughts on my concern.

• I wonder what the registration practice is concerning the clinical management after PROM or preterm PROM with regard to the variables spontaneous vs. planned delivery. Is PROM/PPROM followed by intervention (e.g., prostaglandins, oxytocin) considered induction of labour or spontaneous labour? And is the registration practice uniform across NSW?

• With regard to timing of delivery, on what grounds (e.g., ultrasound scan) is the due date decided?

• I imagine that medical treatment of GDM is a part of the explanatory variables? Also, it would be very informative if the medical treatment is sub-specified as the recommendations of specific medications vary internationally.

• With regard to the sensitive matter of race and ethnicity, country of birth is mentioned as an explanatory variable, yet I anticipate that including ethnicity as an additional variable will contribute with valuable information given the diverse ethnic population in Australia. Especially with regard to generalisability of the study findings to other populations worldwide. However, I might potentially be misunderstanding the Aboriginality variable and ethnicity might already be addressed in the study protocol?

• Finally, what was the rationale for choosing an 8-fold approach in the K-fold cross-validation?

**Reviewer**	**2**
**Name**	**Cai, Li**

Affiliation **Sun Yat-Sen University, Department of Maternal and Child Health, School of Public Health**

Date **11-May-2024**

COI **None.**

This is a large cohort based on a population of pregnant women, and the study aims to investigate the relationship of gestational diabetes with adverse outcomes and to provide guidance for the diagnosis and management of GDM.

The protocol is well-written, the data are of high quality, and the data analysis is diverse, including risk prediction models and logistic regression models. I have just a few comments as follows.

Major comments:

1. Limitations of previous studies can be summarized to highlight the innovations of this study.

2. All women who give birth during the study period are included. However, some pregnant women in the included population may have serious diseases that even affect the outcome. Are there inclusion and exclusion criteria for the study population?

3. The protocol has shown the definitions of GDM and PGDM. It's better to add some details of statistical analysis of the two populations. Such as whether to segregate the PGDM population from the GDM population.

4. In the hospital variation analyses, directed acyclic graphs are used to understand causal effects. Is it convenient to add these graphs in the protocol?

5. Sensitivity analysis is recommended in the data analysis to test the robustness of the results.

Minor comments:

1. “The new criteria used new glucose diagnostic cut-offs, and changed diagnosis of GDM to a one-step universal fasted 75g OGTT.” Please provide the specific cut-off values of the new criteria.

2. In analysis of aim 2, why you decided to choose women with diet-controlled GDM with a singleton pregnancy and cephalic presentation as study population?

3. Please give detailed information of confounding factors in analysis plan.

VERSION 1 - AUTHOR RESPONSE

Reviewer: 1
Dr. Maria Hornstrup Christensen, Odense University Hospital
Comments to the Author:
General comment

The study protocol represents a thorough and ambitious piece of work. The study will contribute with novel, highly relevant and valuable insights for clinical practice that will further qualify and optimise the shared decision-making and care for women with GDM and their offspring. As such, I anticipate that the results from the study will be eagerly awaited by all involved in GDM clinical management as well as in GDM research.
The interdisciplinary author group constitutes an important strength for the designing and the conduct of the study and must be praised.

I have the following specific comments and questions to the study protocol:
• For clarification, I suggest that the abstract states that Aim 2 addresses women with diet-controlled GDM (as noted on page 10). In addition, I recommend that it is mentioned in the overview of the aims on page 5.

'Among women with diet-controlled GDM' has now been added to Aim 2 in the main manuscript, and 'diet controlled' has been added in the aims of the abstract so that it now reads: (2) guide optimal birth timing for women with diet-controlled GDM.

• How come the study period is defined as 2016–2020? What is the background for not extending the study period to e.g. 2022/2023?

We would love to include more recent data but none of the authors have access to data beyond 2020. It would take at least 12 months for us to get additional data (and most likely another 3 years to get the pathology data), plus considerable costs for which we have no funding.

• The concept regarding the study population for Aim 1 is not completely clear to me. Does the study population comprise all women both with and without GDM; women undergoing glucose tolerance testing (page 2, l. 23); or women with GDM (page 5, l. 46; page 9, l. 43)? If non-GDM women are included in the study population, how will it be dealt with that 'control women' may have pregestational diabetes (type 1, type 2, other types) and hence contribute with data to the non-GDM group?

The study population for Aim 1 is all women identified as having GDM. The second sentence in the Analysis plan has been extended to clarify this, so that it now reads:

> *The study population, comprising all women in the cohort who have been identified as having GDM, will be randomly divided…*

• How will the possibility of additional pregnancies by each individual woman during the study period be handled? And if the study unit is pregnancy (rather than the individual woman), how will this be accounted for with regard to interdependent data?
Repeated measures logistic regression modelling will be used to account for the non-independence of two or more pregnancies to an individual woman during the study period. We have now clarified this point in the analysis plan so that it now reads:

> *Using the derivation cohort, univariate repeated measures logistic regression modelling will assess the relationship between the primary composite outcome (any of the following: large for gestational age, stillbirth, neonatal death, shoulder dystocia, bone fracture, arm/hand palsy related to birth injury, gestational hypertension or pre-eclampsia) and explanatory variables. Repeated measures logistic regression will take into account the non-independence of two or more pregnancies to the same woman during the study period. Two-way interactions (eg BMI and hyperglycaemia) will be examined, and the collective and individual effect on the multivariate model assessed. Multivariable repeated measures logistic regression will estimate adjusted odds ratios and their 95% confidence intervals.*

• The primary outcome contains adverse outcomes representing very different levels of seriousness (any of: LGA, stillbirth, neonatal death, shoulder dystocia, bone fracture, arm/hand palsy related to birth injury, gestational hypertension or pre-eclampsia). Additionally, 6 of them are primarily related to the offspring and 1 primarily related to the mother. I wonder what the rationale for choosing the specific outcomes included in the primary outcome was and I have some concerns that the interpretation of this outcome will be diffuse and difficult and offer knowledge with somewhat questionable clinical applicability? I would like to hear the authors' thoughts on my concern.

We appreciate the reviewer's concern and agree that the included outcomes represent very different levels of seriousness. We had lengthy discussions about which outcomes to include in the composite outcome and were keen to include only outcomes that were unlikely to have iatrogenic causes, but still clinically significant. Similarly to RCTs that have a 'composite outcome' of outcomes that vary in severity e.g. death/stillbirth vs gestational hypertension, these outcomes remain clinically significant, and were chosen in consultation with obstetric, endocrine and neonatal input.

• I wonder what the registration practice is concerning the clinical management after PROM or preterm PROM with regard to the variables spontaneous vs. planned delivery. Is PROM/PPROM followed by intervention (e.g., prostaglandins, oxytocin) considered induction of labour or spontaneous labour? And is the registration practice uniform across NSW?

PPROM/PROM followed by intervention such as prostaglandins *or oxytocin is considered induction of labour. This practice is uniform across NSW. However, the induction of labour can be considered separately in analysis if required.*

• With regard to timing of delivery, on what grounds (e.g., ultrasound scan) is the due date decided?

The due date is based on the best clinical estimate using ultrasound in the first trimester; if ultrasound and last menstrual period do not agree within five days, then the due date is based on the first trimester ultrasound.

• I imagine that medical treatment of GDM is a part of the explanatory variables? Also, it would be very informative if the medical treatment is sub-specified as the recommendations of specific medications vary internationally.

Antenatal medical treatment of GDM (insulin, oral hypoglycaemic medication) has been added to "Pregnancy risk factors". Unfortunately we do not have information on the specific oral hypoglycaemic medication used.

• With regard to the sensitive matter of race and ethnicity, country of birth is mentioned as an explanatory variable, yet I anticipate that including ethnicity as an additional variable will contribute with valuable information given the diverse ethnic population in Australia. Especially with regard to generalisability of the study findings to other populations worldwide. However, I might potentially be misunderstanding the Aboriginality variable and ethnicity might already be addressed in the study protocol?

Country of birth and Aboriginality are included in our available datasets and hence we are including them as explanatory variables, with country of birth being a proxy for ethnicity. We do not have any additional information on ethnicity, and hence cannot include it.

• Finally, what was the rationale for choosing an 8-fold approach in the K-fold cross-validation?
To describe the k-fold cross validation process, we have used 8-fold as an exemplar for the diagram. From previous experience, 8-fold was a reasonable compromise between computational requirements and prediction error for a similar population size, and is a reasonable starting point for these analysis. The manuscript text has been revised to clarify this, and now reads:

> *Figure 3 provides an illustration of the k-fold approach using an 8-fold as an exemplar of the methodology, where the whole population gets randomly assigned to eight roughly equal groups.*

Reviewer: 2
Dr. Li Cai, Sun Yat-Sen University
Comments to the Author:

This is a large cohort based on a population of pregnant women, and the study aims to investigate the relationship of gestational diabetes with adverse outcomes and to provide guidance for the diagnosis and management of GDM.
The protocol is well-written, the data are of high quality, and the data analysis is diverse, including risk prediction models and logistic regression models. I have just a few comments as follows.

Major comments:
1. Limitations of previous studies can be summarized to highlight the innovations of this study.

In paragraph 4 of the introduction, we have highlighted the results of various clinical studies that have found no evidence of improved maternal or neonatal outcomes after implementation of the WHO recommendations for GDM diagnosis. We have articulated that these findings have informed the current study protocol. To our knowledge, there are no large registry-based studies that have compared maternal and neonatal outcomes for GDM with attention to variation in geographic and hospital-level factors. Therefore, this leads to the innovative and specific study aims and design of the SUGARED study.

2. All women who give birth during the study period are included. However, some pregnant women in the included population may have serious diseases that even affect the outcome. Are there inclusion and exclusion criteria for the study population?

Women with maternal comorbidity will be included as an explanatory variable in studies addressing Aims 1 and 3, but will be excluded from the study addressing Aim 2:

> *Aim 1 – study population excludes women with pre-existing diabetes and multiple births (twins and higher order). Maternal co-morbidities will be included in modelling as explanatory variables.*
> *Aim 2 – study population excludes women with pre-existing medical conditions (Type 1 and 2 diabetes, chronic hypertension, renal, autoimmune, psychiatric, cardiac and respiratory conditions), multiple births (twins and higher order) and pregnancy hypertension as these potentially may influence timing of birth.*
> *Aim 3 - maternal co-morbidities would be included as explanatory variables.*

To clarify this in the manuscript, 'Women with pre-existing comorbidity will also be excluded' has been added to the end of the paragraph with the heading: Analysis plan: Gestational age-specific risk associated with planned birth (Aim 2).

3. The protocol has shown the definitions of GDM and PGDM. It's better to add some details of statistical analysis of the two populations. Such as whether to segregate the PGDM population from the GDM population.

The focus of all of these studies is GDM, and pre-existing diabetes is defined so that this group can either be excluded or used as a comparison.

4. In the hospital variation analyses, directed acyclic graphs are used to understand causal effects. Is it convenient to add these graphs in the protocol?

We appreciate this suggestion of including DAGs in our protocol paper and agree it could be useful. However, inclusion of a DAG at this juncture is not practical. Developing accurate and comprehensive DAGs is an iterative process that requires continuous refinement as more data and insights become available. Prematurely presenting DAGs could limit our ability to adapt our causal models based on actual data characteristics and preliminary findings. It is important that we maintain the flexibility needed for robust causal inference.

5. Sensitivity analysis is recommended in the data analysis to test the robustness of the results.

We intend to undertake sensitivity analyses where appropriate.

Minor comments:
1. "The new criteria used new glucose diagnostic cut-offs, and changed diagnosis of GDM to a one-step universal fasted 75g OGTT." Please provide the specific cut-off values of the new criteria.

The specific cut-off values (fasting plasma glucose ≥5.1 mmol/L, 1 hour plasma glucose ≥10 mmol/L and 2 hour plasma glucose ≥8.5 mmol/L) have been added and an additional reference added [11: International Association of Diabetes and Pregnancy Study Groups Consensus Panel. International association of diabetes and pregnancy study groups recommendations on the diagnosis and classification of hyperglycemia in pregnancy. Diabetes Care *2010;33(3):676–82. doi: 10.2337/dc09-1848.]*

2. In analysis of aim 2, why you decided to choose women with diet-controlled GDM with a singleton pregnancy and cephalic presentation as study population?

The risks for twin and higher order pregnancies are greater than for singleton pregnancies and the optimal timing of birth for multiple pregnancies is likely to be different from that for single pregnancies. Our focus is the optimal timing of birth for low risk women with GDM, as this is the group for whom the benefit of intervention is likely to be the lowest, and who may be at risk of harm from intervention.

3. Please give detailed information of confounding factors in analysis plan.
Similar to the issue with the DAGs, many of the explanatory variables are potentially confounding factors. We will be relying on a-priori knowledge (from the literature as well as clinical expertise) as well as data driven results to arrive at the final set of confounding factors. Having not yet carried out the analyses, it would be premature to have a definitive list of confounders at this stage.
